# Pericytes: The forgotten controllers of a functional blood–brain barrier

**DOI:** 10.1371/journal.ppat.1013145

**Published:** 2025-05-19

**Authors:** Marshall Roedel, Nia Brooks, Tracey J. Lamb

**Affiliations:** Department of Pathology, Emma Eccles Jones Medical Research Building, University of Utah, Salt Lake City, Utah, United States of America; Medical Research Council Laboratory of Molecular Biology, United Kingdom of Great Britain and Northern Ireland

## Introduction

**Fig 1:**
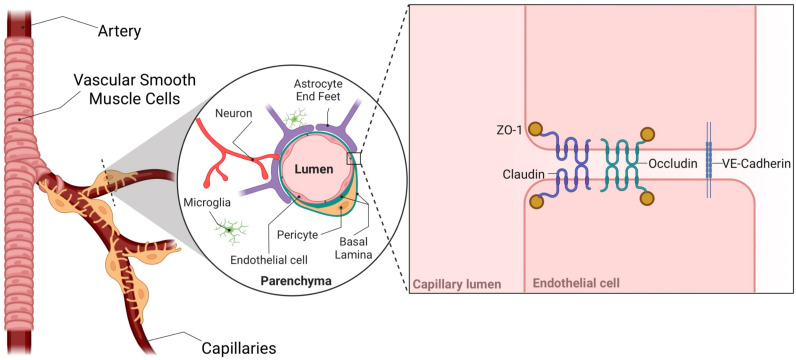
The blood–brain barrier tightly controls the transfer of solutes and cells between the lumen and the parenchyma. Veins and arteries in the brain are tightly wrapped by vascular smooth muscle cells, while the endothelial cells of the capillaries are supported by the other cells of the neural vascular unit, including pericytes astrocytes, neurons, and microglia. Signals received from these cells by endothelial cells induce the expression of tight junction (claudin, occludin) and adherens junction (VE-cadherin) proteins. Homotypic interactions between junction proteins on neighboring endothelial cells keep brain endothelial cells closely adhered to each other and reduce paracellular permeability. Figure created using Biorender.

Neural vasculature exhibits special properties which protect the brain from toxins, pathogens, and immune cell infiltration from the blood. These properties are afforded by the blood–brain barrier (BBB), a semi-permeable membrane separating the blood stream from the brain interstitium. The permeability of the BBB is controlled by the expression of tight and adherens junction proteins by endothelial cells which bind to the corresponding proteins on neighboring endothelial cells to control para-cellular permeability and the careful regulation of transporters and vesicle trafficking to prevent trans-cellular permeability. Signals from other cells, such as pericytes, astrocytes, neurons, and microglia induce these properties in brain endothelial cells and form the neurovascular unit (NVU). An extra-cellular structure call the basal lamina facilitates contacts and the spatial organization between the cells of the NVU (Fig 1). Brain pericytes play a critical role in maintaining BBB integrity and cerebral vascular health. Neurological infection disrupts pericyte function resulting in BBB disruption and this may underpin the correlations of infections with stroke, neurodegeneration, and long-lasting neurological dysfunction [1]. While many *in vitro* studies have elucidated mechanism through which infection of pericytes results in BBB disruption, very few of these findings have been validated *in vivo*. Here we review how pericytes interact with microvascular brain endothelial cells (MBECs) to provide the necessary signals to maintain a healthy and functional BBB. We discuss how infections impair pericyte function and review available models which are available to study the inflammatory modulators of pericyte function during infection.

## What are brain pericytes?

Mural cells surround and interact with the abluminal endothelial cell lining of blood vessels controlling blood flow. They fall into two categories: vascular smooth muscle cells (VSMCs) which ensheathe veins, arteries venules, and arterioles and pericytes which ensheath capillaries. There are three kinds of pericytes based on morphology: (1) ensheathing, which have processes that wrap around the vessel, (2) mesh, which have stellate processes, and (3) thin strand, which have two processes that run parallel to the capillary on either side. Mesh and thin strand pericytes are located on the capillaries, whilst ensheathing pericytes are found on the pre-capillary arterioles and the post-capillary venules, found respectively upstream or downstream of the capillaries [2]. In addition to controlling blood flow through vessel contraction, brain pericytes regulate angiogenesis, are multipotent giving rise to neural and vascular lineage cells in response to cerebral insult, and confer barrier properties onto brain endothelial cells [3,4].

## How do pericytes maintain blood–brain barrier function during homeostasis?

Brain endothelial cells receive multiple signals from adhered pericytes to maintain barrier integrity via molecules that include transforming growth factor beta (TGF-β), notch ligands, and angiopoietin (Angpt) 1 [5]. Pericytes are guided to endothelial cells through the interaction of platelet-derived growth factor receptor beta (PDGFRB) with endothelial-expressed PDGFB. In response endothelial cells mediate adhesion via Smad4-mediated upregulation of the adhesion molecule N-cadherin [6]. The genetic deletion of PDGFB removes pericyte adherence to brain MBECs, and is embryonically lethal [7]. Homozygous expression of a mutation of the heparan sulfate proteoglycan-binding motif in PDGFRB (PDGFRB^ret/ret^) results in an incomplete reduction of pericyte adherence and allows survival to adulthood but with increased permeability. There is also a higher occurrence of sprouting tip cells, suggesting increased initiation of angiogenesis in the absence of optimal binding of PDGFB to PDGFRB [7]. Single-cell RNA sequencing of brain endothelial cells from the PDGFRB ret/ret mice show a generalized increase in Angpt2, which historically has been thought to destabilize endothelial barriers by down-regulating tight and adherens junction proteins. However an endothelial cell-specific knockout of Angpt2 in the PDGFTB^ret/ret^ model does not decrease leak but increases it, suggesting a protective role.

## How is brain pericyte activity modulated during infection?

While pericytes have been shown to be vulnerable to infection by the bacteria *Bartonella henselae*, and respond to lipopolysaccharide (LPS), the pericyte response to bacterial, fungal, or parasitic infection, and its effect on BBB integrity, is poorly studied. Most of the information available on how pericytes respond to infection centers on the viral infection. Indeed pericytes may themselves be directly vulnerable to a number of viral infections. Primary human pericytes can be infected by human immunodeficiency virus (HIV) [8], cytomegalovirus [9], and Japanese encephalitis virus (JEV) [10]. Severe Acute Respiratory Syndrome Coronavirus-2 (SARS-CoV2) cannot replicate in human-induced pluripotent stem cell-derived brain-like pericytes [11], though the SARS-CoV2 spike protein has been shown to affect pericyte constriction function through interaction with pericyte angiotensin converter enzyme-2, a critical receptor used by SARS-CoV2 to enter cells [12]. While primary pericytes are vulnerable to these viruses, it is not known to what degree, or under what conditions, these viruses can cross the endothelium to interact with brain pericytes. Simian immunodeficiency virus (SIV) viral particles can be detected in pericytes in areas of fibrinogen extravasation in sections of macaque brains, though it is unclear if the barrier dysfunction preceded or is a result of pericyte infection [13]. JEV can infect brain endothelial cells *in vitro*, but this in itself does not disrupt barrier function; infection of a coculture of brain endothelial cells and pericytes does result in barrier disruption [14] suggesting that *in vivo* JEV may infect endothelial cells before the infection is spread to pericytes, in turn mediating pathogenesis.

**Fig 2:**
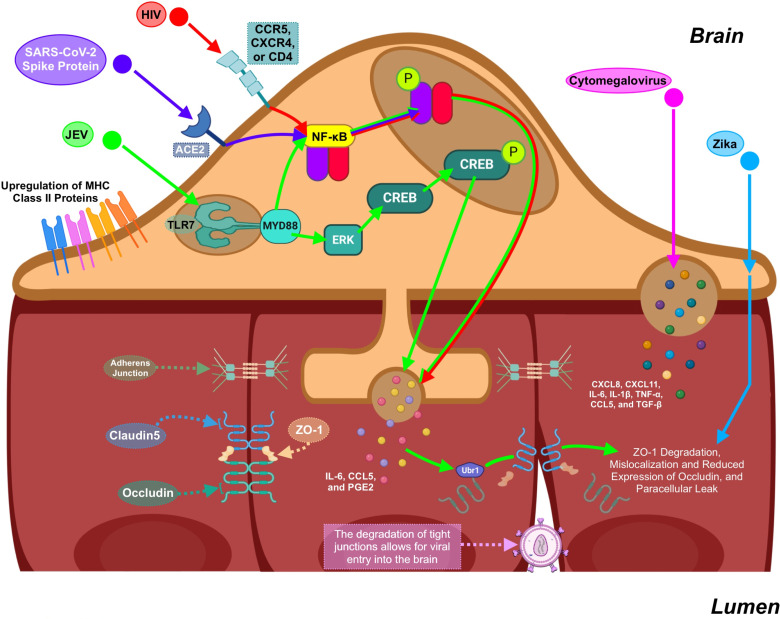
Virus-infected pericytes impair brain endothelial cells and BBB function. Brain pericytes are susceptible to infection by HIV, JEV, cytomegalovirus, and Zika virus. HIV enters pericytes through CCR5, CXCR4, or CD4. SARS-CoV-2 spike protein can trigger pericytes through the ACE-2 receptor. Once in the cells, viruses activate the NF-κB pathway. JEV has also been shown to activate the ERK/CREB pathway through TLR7 and MyD88. NF-κB activity induces the secretion of cytokines such as IL-6, CCL5, and PGE2, which increase the permeability of the brain endothelium. IL-6 induces permeability by activiating UBR1 in brain endothelial cells, which marks the tight-junction protein occludin for degradation. While the invasion receptors and downstream pathways are not known, cytomegalovirus and zika virus can also reduce endothelial BBB function through the secretion of cytokines such as CXCL8, CXCL11, IL-6, IL-1β, TNF-α, CCL5, and TGF-β. HIV, Human Immunodeficiency Virus; JEV, Japanese Encephalitis Virus; SARS-CoV-2, Severe Acute Respiratory Syndrome Coronavirus-2; CCR-5, C-C chemokine receptor 5; CXCR4, C-X-C chemokine receptor type 4; CD4, cluster of differentiation 4; ACE-2, angiotensin converter enzyme 2; NF-κB, nuclear factor kappa B; ERK, extra-cellular signal-regulated kinase; CREB, cyclic adenosine monophosphate response element binding protein; TLR7, toll-like receptor 7; MYD88, Myeloid differentiation primary response protein 88; IL-6, interleukin 6; CCL-5, chemokine (C-C motif) ligand 5; PGE2, prostaglandin E2; UBR1, ubiquitin protein ligase E3 component n-recognin 1; CXCL8, interleukin-8; CXCL11, C-X-C motif chemokine ligand 11; IL-1β, interleukin-1 beta; TNF-α, tumor necrosis factor alpha; TGF-β, Transforming growth factor beta. Figure created using Biorender.

Though the entry receptors through which different viruses infect brain pericytes are distinct, the effects of infection by different viruses converge in the activation of the NF-κB pathway [10,15] (Fig 2). HIV can downregulate the expression of Sirtuin-1, which results in decreased de-acetylation of the NF-κB P65 subunit, in turn leading to NF-κB activation [8]. NF-κB activation in pericytes leads to morphological changes in pericytes, the implication of which is poorly understood. Cultured human primary brain pericytes respond to SARS-CoV2 spike protein by upregulating α-Sma and shifting from a globular morphology to a more branched morphology [15]. Netrins, a family of proteins that have been shown to increase endothelial proliferation, vascular branching, and vessel density, are also upregulated by pericytes in response to SIV in macaques [13]. This response may be initiated to try to maintain any breach in the barrier.

Pericytes also have the ability to respond to signals from the immune system indicating an infectious disease is present. The role of type 1 interferon, the principal cellular response to viral sensing, is poorly described but likely to play a role in pericyte responses to viral infection given the role this cytokine has in BBB disruption in non-infectious disease settings like neurodegeneration [16]. Once activated during infection, either directly by viral invasion or in response to immune signals indicative of an infection such as type 1 interferons, the pericyte cross-talk with MBECs can disrupt barrier function. Although poorly understood, induction of vascular endothelial growth factor (VEGF) and Notch3, both of which induce angiogenesis [17], can disrupt endothelial cell junction protein maintenance via endothelial-expressed VEGFR and Jag3 respectively.

## Do pericytes directly initiate or participate in immune responses?

Pericytes may have some capacity to directly mobilize an immune response on some level. The viral entry receptors used by viruses to infect pericytes include TLR7 (JEV; [14]) and CD4, CXCR4, and CCR5 (HIV; [18]) (Fig 1). Pericytes can also respond to LPS via TLR4 [19]. Collectively this suggests that pericytes may use pattern recognition receptors and respond to immunological cues in a manner similar to innate immune cells. One effect of viral activation of NF-κB in pericytes is production of cytokines such as IL-6 and IL-β [10,18]. *In vitro* exposure of MBEC to viral infection with the addition of IL-6 blocking antibodies suggests there could be a contribution of these cytokines to the disruption of BBB integrity [14]. In experiments using JEV [14] or HIV [18] such a finding has been correlated with a decrease in ZO-1 expression by brain endothelial cells when they are co-cultured with JEV-infected pericytes or exposed to media from infected brain pericytes, but not when endothelial cell mono-cultures are infected. For cultures with JEV, this finding was dependent on the activity of the ubiquitin-proteasome, which is activated by IL-6R signaling [14]. There may also be an influence of pericytes on chemokine production either directly or via their interaction with MBEC.

The increase in leukocyte extravasation into the brain in mouse models where pericytes have been depleted suggests a contribution of pericytes to preventing leukocyte extravasation during steady state [7] albeit this could be via a direct or indirect effect. Beyond innate immune functions, data showing that pericytes have the capability to present alloantigen to T effector cells in the context of transplantation [20] suggest that the immunological capabilities of pericytes may be more extensive than currently appreciated.

## What study systems are available for dissecting pericyte responses to infection *in vivo*?

A major challenge for studying the brain pericyte response to infection *in vivo* has been the lack of identification of pericyte-specific molecular markers (Table 1). Many proteins traditionally used as markers for pericytes, such as PDGFRB, T-Box transcription factor 18 (TBX-18), and alanine aminopeptidase (ANPEP, or CD13) are also expressed in VSMCs [21]. Due to their similar functions it is perhaps initially unnecessary to distinguish the individual contributions of pericytes and VSMCs to BBB health in the context of infection. In addition to overlap with VSMCs, there is overlap with oligodendrocyte precursors and some perivascular macrophages in the expression of neural/glial antigen 2 [21]. The situation is further complicated by the fact that some pericyte markers become upregulated by other cells in a disease context. As an example, PDGFRB can be expressed by astrocytes. α-smooth muscle actin (α-Sma), which is expressed in VSMCs and the ensheathing pericytes of the pre-capillary arterioles in steady-state, is expressed by other pericytes in the context of inflammation [21]. Recently, a review of several pericyte sequencing datasets identified atpase 13a5 (ATP13a5) as a marker specific to pericytes, and an inducible cre mouse with Atp13a5 as its driver has been created [22]. While this tool may be valuable for studying pericyte-specific contributions to BBB health in disease contexts, the expression of Atp13a5 by other cells in non-steady state conditions has not been fully explored.

**Table 1:** Markers of pericytes and their expression on other cells of the brain.

Pericyte marker	Brain cells which express at steady state	Brain cells which express during infection/inflammation	Mouse model
PDGFRβ	Pericytes, Smooth Muscle Cells	Pericytes, Smooth Muscle Cells, Neurons, Astrocytes	B6.Cg-Tg(Pdgfrb-cre/ERT2)6096Rha/J, PDGFRB^ret/ret^
α-SMA	VSMCs, Ensheathing Pericytes	VSMCs, More general pericyte expression	B6(Cg)-Tg(Acta2-cre/ERT2)1Ikal/J
NG2	Pericytes, Oligodendrocyte Precursors, Perivascular Macrophages,	Pericytes, Oligodendrocyte Precursors, Perivascular Macrophages	B6.Cg-Tg(Cspg4-cre/Esr1*)BAkik/J, Tg(Cspg4-DsRed.T1)1Akik/J
Anpep (CD13)	Pericytes, VSMCs	Pericytes, VSMCs	
Tbx-18	Pericytes, VSMCs	Pericytes, VSMCs	Tg(Tbx18-icre)3Fech/J
Atp13a5	Pericytes	?	Atp13a5-2A-CreERT2-IRES-tdTomato

### Perspectives

Pericytes play important roles in vascular health, but they remain an understudied cell type. The effect of pericyte infection on the functional control of blood flow and angiogenesis is poorly understood. The capabilities and scope of pericytes as immune cells are an area for future research which will inform the field of neuroinflammation. A better understanding of the relationship between pericyte infection and function may reveal targets for novel therapies that improve brain vascular health and protect against common neurological disorders and should be a promising focus for future research.
